# Patient characteristics and in‐hospital complications of subcutaneous implantable cardioverter‐defibrillator for Brugada syndrome in Japan

**DOI:** 10.1002/joa3.12234

**Published:** 2019-09-16

**Authors:** Iwanari Kawamura, Mikio Nakajima, Takeshi Kitamura, Richard H. Kaszynski, Rintaro Hojo, Hiroyuki Ohbe, Yusuke Sasabuchi, Hiroki Matsui, Kiyohide Fushimi, Seiji Fukamizu, Hideo Yasunaga

**Affiliations:** ^1^ Department of Cardiology Tokyo Metropolitan Hiroo Hospital Tokyo Japan; ^2^ Emergency and Critical Care Center Tokyo Metropolitan Hiroo Hospital Tokyo Japan; ^3^ Department of Clinical Epidemiology and Health Economics School of Public Health The University of Tokyo Tokyo Japan; ^4^ Data Science Center Jichi Medical University Tochigi Japan; ^5^ Department of Health Policy and Informatics Tokyo Medical and Dental University Graduate School of Medicine Tokyo Japan

**Keywords:** Brugada syndrome, complication, subcutaneous implantable cardioverter‐defibrillator, transvenous implantable cardioverter‐defibrillator, ventricular fibrillation

## Abstract

**Background:**

Clinical features and complications of subcutaneous implantable cardioverter‐defibrillator (S‐ICD) implantation for Brugada syndrome have not been well studied.

**Methods:**

We used the Japanese Diagnosis Procedure Combination database to retrospectively investigate patients who had undergone ICD implantation between April 2016 and March 2017. We compared the characteristics and in‐hospital complications of patients with Brugada syndrome implanted with S‐ICD or transvenous (TV)‐ICD.

**Results:**

We extracted 3090 patients who received ICD implantation. Among them, we identified 278 Brugada patients. The mean age was 43 ± 14.4 years and 262 (94%) were male. Of these 278 patients, 136 (49%) received S‐ICD and 142 (51%) received TV‐ICD. TV‐ICD recipients had a history of atrial fibrillation more frequently compared with S‐ICD recipients. The median (interquartile range) of length of hospital stay was not significantly different between patients with S‐ICD and TV‐ICD (13 days [10‐20.5] vs 12 days [10‐18], respectively). The prevalence of in‐hospital complications after ICD implantation was similar between the two groups. There were no patients with cardiac tamponade, hemothorax, pneumothorax, cardiovascular event, stroke, and death following the procedure during hospitalization in either group.

**Conclusions:**

Short‐term safety of S‐ICD implantation may be identical to that of TV‐ICD. Large prospective studies are warranted to compare the effects and long‐term safety of S‐ICD compared with TV‐ICD.

## Abstract 2

In the present study, we aimed to compare the patient characteristics and in‐hospital complication of patients with Brugada syndrome implanted with subcutaneous implantable cardioverter‐defibrillator S‐ICD (S‐ICD) or transvenous (TV)‐ICD. From the Japanese nationwide inpatient database, we extracted 3090 patients who received ICD implantation. The present study is the first to describe patient characteristics and in‐hospital complication of TV‐ICD and S‐ICD in patients with Brugada syndrome in Japan.

## 1 INTRODUCTION

Brugada syndrome is a heritable arrhythmia syndrome that increases an individual's risk for sudden cardiac death attributable to ventricular fibrillation (VF).^1^ Most cardiac events occur at night during the sleep of apparently healthy subjects, especially Southeast Asian middle‐aged men.^2^ The only proven and effective therapeutic strategy for the prevention of sudden cardiac death in high‐risk Brugada syndrome is an implantable cardioverter‐defibrillator (ICD).^2^, ^3^, ^4^ It is important to recognize that ICDs are associated with complications, especially in young active individuals.^5^

A subcutaneous implantable cardioverter‐defibrillator (S‐ICD) has been adopted as an alternative to transvenous (TV)‐ICD.^6^, ^7^ S‐ICD does not require placement of leads directly into the heart transvenously; hence these devices could avoid the complications related to the use of TV‐ICD leads.^8^, ^9^ Moreover, since the incidence of lead injury increases over time after TV‐ICD implantation,^5^, ^10^ use of S‐ICDs would be expected to avoid troubles concerning cardiac leads, especially in younger patients without organic heart disease. Most patients with Brugada syndrome do not require the pacing function of TV‐ICD for bradycardia or sustained ventricular arrhythmia.^2^ Although S‐ICD may be preferential for Brugada syndrome, inappropriate shocks due to T‐over sensing and atrial high rate episodes would be the most common issue associated with S‐ICD systems.^11^, ^12^ While a larger scale comparative study on S‐ICD versus TV‐ICD has been previously conducted, there currently are no descriptive studies comparing the two as they relate specifically to use in Brugada patients.^13^

Although the comparison of effects and safety between TV‐ICD and S‐ICD in Brugada syndrome are critical points to consider in the clinical setting, patient characteristics and short‐term complications of TV‐ICD and S‐ICD in patients with Brugada syndrome remain unknown. The aim of the present study was to elucidate the clinical features and complications of S‐ICD compared with TV‐ICD implantation in patients with Brugada syndrome in Japan, using a nationwide inpatient database.

## 2 METHODS

The Institutional Review Board of the University of Tokyo approved this study. The requirement for informed consent was waived because of the anonymous nature of the data.

### 2.1 Data source

The present study was a descriptive study using the Japanese Diagnosis Procedure Combination inpatient database. The nationwide database contains discharge abstracts and administrative claims data from more than 1200 acute‐care hospitals in Japan. The database includes individual data on following: age; sex; diagnoses; comorbidities on admission; complications after admission; procedures performed during hospitalization; drugs and devices used during hospitalization; date of admission and discharge; body mass index at admission calculated by height and weight; and discharge status (discharge to home, discharge to other facility, and in‐hospital death). These diagnoses are coded using the International Classification of Diseases, Tenth Revision codes (ICD‐10) with text data entered in Japanese. Several studies analyzing this database have been reported in the cardiovascular research field previously.^14^, ^15^

### 2.2 Patient selection

We retrospectively extracted patients who underwent ICD implantation discharged from April 2016 to March 2017 with Brugada syndrome. This study did not include the procedures of re‐implantation of ICD. Owing to the approval date for commercial use of S‐ICD in Japan, this study included the data from April 2016. The diagnosis of Brugada syndrome was identified using Japanese text data in ICD‐10 code, I490.

### 2.3 Variables and outcomes

Patient characteristics included age, sex, emergency admission, and structural heart disease (SHD). We defined SHD as the following: ischemic heart disease (ICD‐10 codes, I20–25); diastolic cardiomyopathy (I420); hypertrophic obstructive cardiomyopathy (I421); non‐obstructive hypertrophic cardiomyopathy (I422); other cardiomyopathy (I423, I424, I425, I426, I427, I428, I429, I43); hypertensive heart disease (I11, I13); congenital heart disease (Q2); sick sinus syndrome (I495); 2nd degree atrioventricular block (I441); complete atrioventricular block (I442); and other and unspecified atrioventricular block (I443). We also evaluated oral medications and other comorbidities such as diabetes mellitus (E100‐149)^16^ or renal failure requiring renal replacement therapy.

The outcomes were length of hospital stay and in‐hospital postprocedural complications. Complications included the following: accidental lead removal requiring lead extraction after ICD implantation; critical bleeding defined by the necessity of blood transfusions; cardiac tamponade necessitating pericardiocentesis, open chest surgery, open chest removal of hematoma, pericardial sutures, and pericardial incision; pneumothorax or hemothorax requiring thoracic drainage; heart failure diagnosed after admission (I50); stroke diagnosed after admission (I630‐635, I638, I639); and postprocedural use of mechanical circulatory support (intra‐aortic balloon pumping or extracorporeal membrane oxygenation). We compared patient characteristics and in‐hospital complications of patients with Brugada syndrome embedded with S‐ICD or TV‐ICD.

In addition to the above, we compared the length of hospital stay in patients admitted under emergency versus planned conditions. The length of hospital stay in patients placed under emergency admission was assumed to be longer in comparison with patients admitted under planned admission due to the comparatively severe status on admission of the former.

We also compared total cost during the hospital stay in both groups (106 yen = 1 US dollar).

### 2.4 Statistical analysis

Values of categorical variables were reported as numbers and percentage, and values of continuous variables were reported as mean with standard deviation (SD) or median with interquartile range (IQR). Continuous variables were compared using Wilcoxon rank sum tests and categorical variables were compared using Fisher's exact tests or chi‐squared test. All analyses were performed using Stata/ MP 14 (StataCorp).

## 3 RESULTS

### 3.1 Patient characteristics

We extracted 3090 patients who received ICD implantation. Among them, we identified 278 Brugada patients. Table 1 summarizes the patient characteristics in this study. The mean age was 43 ± 14.4 years and 262 (94%) were male. Of these 278 patients, 136 (49%) received S‐ICD and 142 (51%) received TV‐ICD. Over 20% of patients were aged >60 years in both groups. There was no significant difference in age and body mass index between the two groups. Patient characteristics were similar between the S‐ICD and TV‐ICD groups with regard to diabetes mellitus (9 vs 11), hypertension (23 vs 23), renal replacement therapy (2 vs 3), and emergency admission (28 vs 34). History of atrial fibrillation was more frequent in TV‐ICD recipients compared with S‐ICD recipients (7 vs 17). Bradycardia and VT displayed a more frequent tendency in TV‐ICD recipients compared with S‐ICD recipients; however, there was no statistical difference.

**Table 1:** Characteristics of patients with Brugada syndrome who received implantable cardioverter‐defibrillator

Variables^a^	All patients (n = 278)	S‐ICD (n = 136)	TV‐ICD (n = 142)	*P*
Age, years, mean ± SD	43 ± 14.4	43 ± 14.0	45 ± 14.9	.51
≤20 years	5 (1.8)	2 (1.5)	3 (0.7)	.96
20‐60 years	213 (76.6)	104 (76.5)	109 (76.8)	
>60 years	60 (21.6)	30 (22.1)	30 (21.1)	
Male	262 (94.2)	134 (98.5)	128 (90.1)	.004
BMI at admission, kg/m^2^				
<18.5 (underweight)	24 (8.6)	10 (7.4)	14 (9.9)	.11
18.5‐24.9 (normal weight)	189 (68.0)	91 (66.9)	98 (69.0)	
25.0‐29.9 (overweight)	55 (19.8)	32 (23.5)	23 (16.2)	
≥30.0 (obese)	5 (1.8)	3 (2.2)	2 (1.4)	
Missing	5 (1.8)	0 (0)	5 (3.5)	
Comorbidities at admission				
Structural heart disease	3 (1.1)	1 (0.7)	2 (1.4)	1.00
Atrial fibrillation	24 (8.6)	7 (5.1)	17 (12.0)	.043^b^
Ventricular tachycardia	17 (6.1)	5 (3.7)	12 (8.5)	.16
Sick sinus syndrome	9 (3.2)	4 (2.9)	5 (3.5)	1.00
Atrioventricular block	12 (4.3)	3 (2.2)	9 (6.3)	.16
2nd degree atrioventricular block	3 (1.2)	1 (0.7)	2 (1.4)	1.00
Complete atrioventricular block	1 (0.4)	1 (0.7)	0 (0)	.98
Other type of atrioventricular block	8 (2.7)	1 (0.7)	7 (4.9)	.08
Hypertension	46 (16.5)	23 (16.9)	23 (16.2)	.87^b^
Diabetes mellitus	20 (7.2)	9 (6.6)	11 (7.7)	.72^b^
Renal replacement therapy	5 (1.8)	2 (1.5)	3 (2.1)	1.00
Emergency admission	62 (22.3)	28 (20.6)	34 (23.9)	.50^b^
Cardiac arrest on arrival	7 (2.5)	1 (0.7)	6 (4.2)	.13

Abbreviations: BMI, body mass index; S‐ICD**,** subcutaneous implantable cardioverter‐defibrillator; TV‐ICD, transvenous implantable cardioverter‐defibrillator.

a Data were shown as number (%) unless otherwise specified.

b Delivered by chi‐squared test.

### 3.2 Outcomes

The median length of hospital stay was not significantly different in patients with S‐ICD and TV‐ICD (13 days [IQR: 10‐20.5] vs 12 days [IQR: 10‐18]). The prevalence of in‐hospital complications after ICD implantation was similar between the groups: lead removal (0 vs 2), blood transfusion (0 vs 1), and heart failure (2 vs 3). There were no patients presenting with cardiac tamponade, pneumothorax, hemothorax, strokes, requiring mechanical circulatory support, and succumbing to death following the procedure in either group (Table 2).

**Table 2:** Outcomes

Outcomes^a^	All patients (n = 278)	S‐ICD (n = 136)	TV‐ICD (n = 142)	*P*
Length of hospital stay, day (IQR)	12 (10‐19)	13 (10‐20.5)	12 (10‐18)	.51
Lead removal	2 (0.7)	0 (0)	2 (1.4)	.52
Blood transfusion	1 (0.3)	0 (0)	1 (0.7)	1.00
Cardiac tamponade	0 (0)	0 (0)	0 (0)	N/A
Heart failure	5 (1.8)	2 (1.5)	3 (2.1)	1.00
Pneumothorax or hemothorax	0 (0)	0 (0)	0 (0)	N/A
Stroke	0 (0)	0 (0)	0 (0)	N/A
Mechanical circulatory support use	0 (0)	0 (0)	0 (0)	N/A
Total cost, US dollar (IQR)	46 057 (43 675‐50 617)	44 306 (43 310‐49 052)	47 092 (45 634‐51 065)	<.001
In‐hospital death	0 (0)	0 (0)	0 (0)	N/A

Abbreviations: IQR, interquartile range; S‐ICD, subcutaneous implantable cardioverter‐defibrillator; TV‐ICD, transvenous implantable cardioverter‐defibrillator.

a Data were shown as number (%) unless otherwise specified.

Patients placed under emergency admission had a significantly longer hospital stay compared with those under planned conditions (11 days [IQR: 9‐15] vs 23.5 days [IQR: 17‐33], *P *< .001).

Regarding total cost during the hospital stay, we observed lower costs in the S‐ICD group than that in the TV‐ICD group (44 306 US dollars (IQR: 43 310‐49 052) vs 47 091 [45 634‐51 065], *P* < .001).

## 4 DISCUSSION

The present study is the first to describe patient characteristics and in‐hospital complications associated with TV‐ICD and S‐ICD in patients with Brugada syndrome in Japan. Approximately half of the recipients underwent S‐ICD and the short‐term safety of S‐ICD implantation was identical to that of TV‐ICD.

Although there are some descriptive studies comparing patient characteristics and short‐to‐midterm complications of S‐ICD with TV‐ICD implantation,^13^, ^17^ there are no data specifically targeting Brugada patients. As opposed to earlier studies, the patient characteristics in the present study were skewed toward a younger demographic, fewer comorbidities, and a higher percentage of males who are inherent to Brugada syndrome. Despite a relatively benign prognosis associated with senior Brugada patients,^18^ approximately 20% of ICD recipients in the present study were over the age of 60 in both groups. The prevalence of in‐hospital complications of S‐ICD was similar to those in previous studies.^13^, ^19^

Previous studies have documented a reduced risk of lead complication in S‐ICD patients.^19^, ^20^ The pacing function for bradycardia and sustained monomorphic VT is not warranted for the vast majority of Brugada patients.^2^ Considering the relatively young age of Brugada patients, S‐ICD would be preferential mode of treatment given the reduced risk of developing long‐term complications such as lead infection, venous obstruction, and lead dysfunction. On the opposite end of the spectrum, an inappropriate shock delivered in response to supraventricular tachycardia and T‐over sensing is one of the primary issues associated with S‐ICD use in Brugada patients.

There currently is neither a consensus nor a specific set of guidelines indicating which type of ICD is recommended for Brugada patients. This study showed that roughly half of Brugada patients had underwent S‐ICD implantation in Japan. For patients with a history of atrial fibrillation, bradycardia, and VT, TV‐ICD displayed a tendency to be used at a higher frequency than S‐ICD. There is no significant baseline difference between the groups with regard to the prevalence of renal replacement therapy or diabetes mellitus—both of which would increase the risk of infection.

The length of hospital stay in both groups is much longer than a previous descriptive study conducted in the United States.^13^ There were no severe complications documented in the short term and therefore the present data passively suggest that the length of hospital stay can be safely shortened in Japan.

### 4.1 Study limitations

This study had several limitations. First, it was a retrospective study based on the secondary use of administrative claims datas. Diagnoses based on such sources are generally less well‐validated than those in prospectively collected data based on predefined diagnostic criteria. A previous study which analyzed 16 diseases using the ICD‐10 code in the same database showed the specificity of diagnoses was found to exceed 96%, whereas their sensitivity was limited to a range between 50% and 80%. The specificity and sensitivity of procedures were found to exceed 90%.^21^ Second, a number of complications such as the prevalence of inappropriate therapy or failure to convert ventricular fibrillation could not be evaluated. Third, clinical data such as electrocardiogram, family history, and past history of ventricular fibrillation were not available from the database. Furthermore, we could not obtain the clinical reason why the patient with sick sinus syndrome or atrioventricular block underwent S‐ICD implantation. Forth, we cannot clarify the single leads or dual leads for TV‐ICD. Fifth, the patients cannot be followed‐up after discharge and therefore the long‐term outcome and effectiveness remain unknown. Finally, although we used a nationwide cohort, this study was conducted in a single country; however, given the prevalence of Brugada syndrome in the Asian population, the limited focus on the Japanese population is considered a negligible setback. Large prospective studies are required to compare the effects and long‐term safety of S‐ICD compared with TV‐ICD.

## 5 CONCLUSION

S‐ICD is widely adopted in patients with Brugada syndrome in Japan. The short‐term safety of S‐ICD implantation may be identical to that of TV‐ICD; however, a large‐scale prospective study is warranted to compare the effects and safety of S‐ICD compared with TV‐ICD.

## CONFLICT OF INTEREST

The authors declare no conflict of interests for this article.
